# Can administrative data be used for a national register of hospitalised stroke patients? A New Zealand validation study

**DOI:** 10.1016/j.lanwpc.2025.101768

**Published:** 2026-01-07

**Authors:** Marine Corbin, Hayley J. Denison, Jeroen Douwes, Mina Whyte, Stephanie G. Thompson, Matire Harwood, Alan Davis, John N. Fink, P. Alan Barber, John H. Gommans, Dominique A. Cadilhac, William M. Levack, Harry McNaughton, Joosup Kim, Valery L. Feigin, Anna Ranta

**Affiliations:** a Research Centre for Hauora and Heath, Massey University, New Zealand; b Department of Medicine, University of Otago Wellington, New Zealand; c Department of General Practice and Primary Health Care, University of Auckland, New Zealand; d Department of Medicine, Whangarei Hospital, New Zealand; e Department of Neurology, Christchurch Hospital, New Zealand; f Department of Medicine, Hawke's Bay Hospital, New Zealand; g Department of Medicine, School of Clinical Sciences, Monash University, Clayton, VIC, Australia; h Medical Research Institute of New Zealand, New Zealand; i National Institute for Stroke and Applied Neurosciences, School of Clinical Sciences, Auckland University of Technology, New Zealand; j Department of Neurology, Wellington Hospital, New Zealand

**Keywords:** Hereditary cancer predisposition syndromes, Genetic panels, Personalized medicine

## Abstract

**Background:**

Using community-based incidence studies and clinical registries to assess stroke care and outcomes is resource intensive and often geographically limited. Linked administrative data are lower-cost and wider-reaching, but potentially less accurate and complete. This study compared administrative data to national hospital-based study data to assess whether administrative data represents a valid alternative.

**Methods:**

We linked and compared data from the REGIONS Care Study, a New Zealand nationwide observational study, with administrative data from Statistics New Zealand’s Integrated Data Infrastructure (IDI). Sensitivity, specificity, positive predictive value, and Cohen’s kappa coefficient were used to assess case identification, risk factors, post-stroke outcomes, and interventions as applicable. Additional audits explored the validity of IDI ‘true false positives.’

**Findings:**

From May to July 2018, 1719 patients with stroke were captured in REGIONS Care and 1833 in the IDI. Using REGIONS Care as the reference standard, the sensitivity of the IDI for stroke case identification was 83% and the positive predictive value 77%. There were 300 false-negatives and 414 false positives. The audit of two hospitals showed that some cases identified in IDI but excluded by REGIONS were actual strokes. For stroke risk factors, the IDI showed high sensitivity and specificity for diabetes (93% and 91%, respectively), atrial fibrillation (87% and 90%), and smoking (71% and 97%) but lower specificity for hypertension (61%), and dyslipidaemia (52%). A derived IDI favourable outcome measure showed good agreement with the modified Rankin Scale (sensitivity 88%, specificity 82%, kappa 0.67). The IDI accurately identified post-stroke medication use (sensitivities 81%–94%, specificities 78%–91%) and thrombectomy interventions (sensitivity 88%, kappa 0.91).

**Interpretation:**

The use of administrative data to ascertain stroke cases, risk factors, interventions and outcomes was feasible and compared well with manual hospital data collection making an administrative data based national stroke register possible, although supplementary data collection for comprehensive care evaluation may be required.

**Funding:**

The study was funded by the NZ Health Research Council (HRC 17/037).

**Research in context**

**Evidence before this study**

We searched PubMed for studies that validated the use of administrative data to capture stroke patients, and stroke risk factors and outcomes between 2000 and 2023. The following search terms were used: ((stroke) AND (validation)) AND (“administrative data”). Although we identified several studies assessing the validity of stroke case ascertainment using administrative data, few assessed the validity of stroke risk factors and outcomes. In New Zealand, no previous studies have been conducted to validate administrative data against clinically confirmed stroke cases.

**Added value of this study**

This study made use of de-identified health and social administrative data available from Statistics NZ's Integrated Data Infrastructure (IDI) to validate, for the first time, New Zealand administrative stroke data against clinician-collected data on hospitalised stroke case ascertainment, risk factors, patient outcomes, and stroke interventions. It showed that administrative data, within the context of a well-developed healthcare system with established data systems, provides a valid and efficient alternative to manual, clinician-collected hospital-based stroke data.

**Implications of all the available evidence**

Findings from this study indicate that while some manual data collection will continue to provide additional value in areas not currently well-captured by administrative data, the development of an administrative national virtual stroke register to track hospitalised stroke patients and service performance for health service planning in NZ and potentially other countries with comparable administrative data and health systems is feasible and useful.

## Introduction

The burden of stroke is increasing, and it is now the second most common cause of death and a leading cause of disability worldwide^1^ associated with considerable economic and social burden.^2^ Regular monitoring of incidence, management, and patient outcomes is important to accurately plan and optimise stroke service provision and manage resource allocation. Population-based stroke registries can be used for this but are resource intensive and hospital-based registries may suffer from selection bias. Alternatively, administrative data may be used, with a 2015 systematic review of international studies concluding that data pertaining to the diagnosis of acute stroke are highly predictive of true cases.^3^ However, evidence that administrative data accurately capture stroke risk factors and outcomes is scarce.^4^^,^^5^ In New Zealand (NZ), linked administrative social and health data are available through Statistics NZ, but this is not currently used to assess stroke incidence or outcomes in hospitals, and no studies have been conducted to validate administrative data against clinically confirmed stroke cases.

The aim of this study was to validate routinely collected administrative data against clinician collected data from a national hospital-based observational study for stroke case ascertainment, stroke risk factors, patient outcomes, and post-stroke interventions. We also aimed to assess whether outcomes and risk factors unavailable in administrative data, specifically modified Rankin Scale (mRS) and stroke severity, could be estimated from other routinely collected data.

## Methods

### Source data and participants

We used data from an observational study (Reducing Ethnic and Geographic Inequities to Optimise New Zealand Stroke Care or REGIONS Care) and administrative data from Statistics NZ's Integrated Data Infrastructure (IDI). REGIONS Care is a nationwide study on stroke care access and outcomes involving prospectively collected data from all 28 NZ hospitals caring for patients with acute stroke and associated rehabilitation and community services.^6^ Diagnosis was based on local stroke clinician clinical diagnosis (intracerebral haemorrhage (ICH), cerebral infarction, stroke unspecified type) supported by available imaging, which in NZ consists primarily of CT imaging although MRI is used in selected cases. Where either CT or MRI imaging supported a stroke despite symptom resolution within 24 h the patient would have been classed as having had a stroke. The study was designed to capture all adults with a discharge diagnosis of stroke between 1 May and 31 July 2018. After this date, consecutive patient recruitment continued until hospitals achieved a sample size of ≥150 (thrombectomy centres), ≥100 (all other centres) or until 31 October 2018, whichever occurred first (the sample size of REGIONS Care was determined based on the specific goals of that study (see reference 6) and set prior to developing the current study). Where a person had more than one stroke during the study period, the first was recorded as the index event, and any further events were considered an outcome. The full methods have been described elsewhere.^6^ Study outcomes were collected via telephone interviews.

The IDI is a longitudinal meta-dataset linked at the individual level. It contains de-identified health and social data on individuals and households from NZ Government and non-government administrative and survey data that are continually updated and can provide a longitudinal record for individuals over time.^7^ Stroke cases were identified via hospital discharge data using the International Classification of Diseases codes (ICD-10) (Australian Manual) where I61 = intracerebral haemorrhage (ICH), I63 = cerebral infarction, and I64 = stroke unspecified type.

Two cohorts of patients with stroke aged ≥18 years were selected from the 2017 resident population in the IDI for comparison with REGIONS Care. IDI Cohort 1 focused on stroke case ascertainment and included all first public hospital admissions with stroke ICD codes (I61 = intracerebral haemorrhage (ICH), I63 = cerebral infarction, I64 = stroke unspecified type) as a principal diagnosis from 1 May to 31 July 2018—the period for which REGIONS Care was designed to capture *all* stroke cases. IDI Cohort 2 focussed on risk factors, outcomes and post-stroke interventions, and included all first public hospital admissions with stroke ICD codes as a principal diagnosis during the whole REGIONS Care study period (1 May-31 October 2018) enabling the use of a larger sample size.

### Data linkage

REGIONS Care patients were linked to the IDI and subsequently to IDI cohorts 1 and 2 using encrypted national health index (NHI) numbers after removal of all identifiable information. We selected stroke cases included in both REGIONS Care and IDI by matching both encrypted patients’ IDs and stroke hospital admission dates, ensuring that admission dates did not differ by more than a month.

### Risk factors

Stroke risk factors collected in REGIONS Care included hypertension, dyslipidaemia, diabetes, atrial fibrillation, prior stroke, prior transient ischaemic attack (TIA) and smoking status. These were extracted by clinicians from inpatient progress notes and hospital discharge summaries. Where a clear Yes/No response was not documented a given risk factor's status was classed as ‘unknown.’ IDI definitions for these risk factors were based on discharge diagnoses as coded by hospital coding teams, pharmacy prescription data, and laboratory tests. For a detailed description of the algorithms of how each risk factor was defined in the administrative data set along with ICD-10 codes see Supplementary Table S1.

### Post-stroke outcomes

The main post-stroke outcome assessed in the REGIONS Care data was the mRS, which was dichotomised into favourable outcome (mRS = 0–3, indicating ability to at least ambulate independently) and unfavourable outcome (mRS = 4–6, where 6 = death). In the IDI mRS is not available; instead, we used an alternative composite post-stroke favourable outcome of ‘no death,’ ‘no job loss,’ and ‘no change of residential address.’ (“IDI favourable/not favourable post-stroke outcome”).^8^ Death at follow-up was obtained from the national ‘Births, Deaths, and Marriages’ dataset. Job loss was assessed using tax data from Inland Revenue. Patients not working at the time of stroke were considered as not losing their job as their unemployment was not a consequence of the stroke. Address change was used as a surrogate to indicate discharge to either an aged residential care facility or moving in with family, both indicating sufficient post-stroke disability to warrant significant additional support with activities of daily living. For this, we used the ‘address notification’ dataset, which provides a longitudinal record of address change notifications sourced from multiple agencies (New Zealand Transport Agency, Ministry of Social Development, Ministry of Health, Kāinga Ora (Housing New Zealand), Ministry of Education, Inland Revenue (IRD), Accident Compensation Corporation (ACC), Department of Internal Affairs (DIA)) and updated at least every 6 months. IDI favourable post-stroke outcome at 3 months was compared against mRS = 0–3 and the three components of the IDI favourable outcome were validated separately against the same indicators collected in REGIONS Care. Stroke severity is an important predictor of post-stroke outcomes and as this is not available in the IDI, hospital length of stay (acute) was used instead as a proxy for two severity indicators measured in REGIONS Care: SSV (six simple variable—age, living alone pre-stroke, pre-stroke functional status, normal verbal Glasgow Coma Scale score, ability to lift arms, ability to walk unaided)^9^ ranging from 0 (most severe) to 3 (least severe) measured on admission, and mRS at discharge. We also conducted an additional analysis including both acute and rehabilitation phase of ‘length of stay.’

Secondary outcomes collected in REGIONS Care included recurrent stroke and readmission to hospital within 3 months of the initial stroke. The same outcomes were estimated using data available in the IDI (see Supplementary Table S1 for definitions).

### Post-stroke interventions

Post-stroke interventions included inpatient endovascular thrombectomy and post-discharge outpatient prescription dispensing of anti-platelets, statins, antihypertensive medications and anticoagulants within 3 months after stroke. IDI procedure codes from hospital data to pharmaceutical data were compared with the data collected in REGIONS Care.

### Hospital medical record audit

We conducted a hospital medical record audit seeking further information on patients with a health administrative stroke discharge diagnosis that had not been captured in REGIONS Care, to assess whether these were true ‘false-positives.’ In addition to a local audit by a senior stroke neurologist (AB) or senior stroke nurse specialist, all non-matching cases underwent second review by AR (senior neurologist) for final adjudication. We identified one urban and one non-urban centre with particularly high false-positive rates and identified all stroke (I61, I63, and I64) discharges via local hospital data analytics teams. This represents the same data as identified via IDI, but pre-de-identification to allow local stroke experts to retrieve the hospital medical records comprising admission records, radiology reports, discharge summaries and if required patient progress notes, to confirm or refute the coding team diagnostic allocation submitted to the IDI. In addition, potential reasons for misclassification were noted. We limited this review to usual residents of the district whose hospital we audited and who initially presented to this hospital.

### Statistical analyses

Accuracy of the identification of stroke cases in the IDI cohort was estimated using sensitivity and positive predictive value (PPV) and corresponding asymptotic 95% confidence intervals (formulas provided in Supplementary Table S2) using the REGIONS Care cases as the reference standard. Sensitivity was defined as the proportion of stroke cases in REGIONS Care who were also identified in the IDI, while PPV was defined as the proportion of stroke cases identified in IDI who were also included in REGIONS care. Specificity could not be calculated because true-negatives (non-stroke cases in both the IDI and REGIONS care) were not included as REGIONS Care did not capture non-stroke cases. Comparison of dichotomous risk factors, outcomes and post-stroke interventions between IDI and REGIONS Care was carried out using sensitivity, specificity, and Cohen’s kappa coefficient and corresponding asymptotic 95% confidence intervals. Kappa values greater than 0.60 indicate substantial agreement, 0.21–0.60 indicate moderate agreement, and those 0.20 or lower were considered poor agreement.^10^ Association between hospital length of stay and SSV and mRS categories was estimated using Kruskal–Wallis test. SAS Enterprise Guide v8.3 was used for all analyses.

### Reporting

The IDI confidentiality requirements necessitate that all counts were randomly rounded up or down to the next multiple of three and percentages calculated from the rounded counts. Therefore, the total numbers in each table vary slightly and may not add to 100%. However, the statistical tests were performed on the unrounded counts. In addition, IDI confidentiality requirements also include for all counts under six and associated results to be suppressed and marked as ‘S’ in the tables.

### Ethics approval

Ethics approval was obtained from the Central Region Health and Disability Ethics Committee (17CEN164).^6^

### Role of the funding source

NZ Health Research Council did not have any role in study design, data collection, data analysis, interpretation, or report writing.

## Results

### Stroke case ascertainment

In total, 1719 people were included in the REGIONS Care Study and 1833 people were identified in the IDI as having had a stroke between 1 May 2018 and 31 July 2018 (Cohort 1, Table 1). Of the stroke patients included in REGIONS Care, 1422 (83%) were also identified in the IDI during the same period (Table 2), while 300 (17%) were missed. Four hundred and fourteen patients with stroke were identified in the IDI who had not been included in REGIONS Care. Assuming REGIONS Care was a true representation of all stroke cases, the sensitivity of the IDI for stroke case ascertainment was 83% and the PPV was 77%; this did not vary significantly by ethnicity (Table 2), sex or age group (Supplementary Table S3). Sensitivity and positive predictive value for ICH (I61) and cerebral infarction (I63) were similar, but they were much lower for ‘stroke unspecified’ (I64) (Supplementary Table S3).

**Table 1:** Demographic characteristics and risk factors for all people with a stroke hospitalisation in the IDI between May and July 2018 and all people in the REGIONS study May to July 2018.

Characteristic	IDI cohort		REGIONS cohort	
	n	%	n	%
Total	1833	100	1719	100
Sex (Females)	927	50.6	840	48.9
Age (median, IQR)	77.6 (66,85.6)		77 (66,85)	
*Missing*	*S*	*S*	*S*	*S*
Ethnicity				
NZ European	1425	77.7	1308	75.4
Māori	201	11.0	195	11.3
Pacific	96	5.2	96	5.6
Asian	81	4.6	84	5.1
Other	9	0.5	12	1.7
*Missing*	*24*	*1.3*	*24*	*1.0*
Stroke type				
ICH	237	12.9	219	12.7
Cerebral Infarction	1494	81.5	1395	81.2
Unspecified Stroke	102	5.6	102	5.9
*Missing*	*S*	*S*	*S*	*S*
Hypertension	1485	81.0	1152	67.0
*Missing*	*S*	*S*	*12*	*0.7*
Diabetes	543	29.6	408	23.7
*Missing*	*S*	*S*	*15*	*0.9*
Dyslipidemia	1140	62.2	624	36.3
*Missing*	*S*	*S*	*21*	*1.2*
Atrial fibrillation	588	32.1	471	27.4
*Missing*	*S*	*S*	*15*	*0.9*
Regular smokers	201	11.0	225	13.1
*Missing*	*99*	*5.4*	*21*	*1.2*
Prior stroke	357	19.5	315	18.3
*Missing*	*S*	*S*	*15*	*0.9*
Prior TIA	186	10.1	213	12.4
*Missing*	*S*	*S*	*21*	*1.2*
NZDEP decile				
1–2	222	12.1	N/A	N/A
3–4	363	19.8	N/A	N/A
5–6	354	19.3	N/A	N/A
7–8	459	25.0	N/A	N/A
9–10	438	23.9	N/A	N/A

S = suppressed (<6).

Where “s” features under ‘*Missing*” this means the number of missing values is known but the count was <6 so the exact number could not be reported.

IDI confidentiality rules require for all counts to be randomly rounded up or down to the next multiple of 3 and percentages calculated from the rounded counts. Therefore, total numbers vary slightly between tables and may not add to 100%. Statistical tests were performed on the unrounded counts.

**Table 2:** Number of stroke cases identified in the IDI and in the REGIONS Care study for the period 1 May 2018–31 July 2018 overall and by ethnic group.

Ethnic group	Identified in IDI	Identified in REGIONS	True-positive	False-negative	False-positive	Sensitivity (95% CI)	Positive Predictive Value (95% CI)
All	1833	1719	1422	300	414	83 (81,84)	77 (76,79)
New Zealand European	1425	1308	1098	207	327	84 (82,86)	77 (75,79)
Māori	201	195	162	33	36	83 (78,88)	82 (76,87)
Pacific People	96	96	75	21	21	78 (70,86)	78 (70,86)
Asian	81	84	66	21	18	76 (67,85)	79 (70,87)
Others	9	12	S	6	S	S	S

S = suppressed (<6).

IDI confidentiality rules require for all counts to be randomly rounded up or down to the next multiple of 3 and percentages calculated from the rounded counts. Therefore, total numbers vary slightly between tables and may not add to 100%. Statistical tests were performed on the unrounded counts.

**Figure 1:**
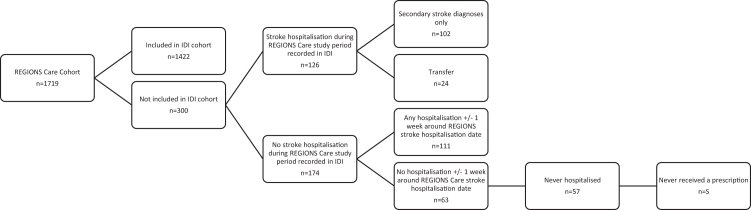
**Explanation of why people who are part of the REGIONS Care cohort did not appear in the IDI cohort as having a stroke during the same period (1st May–31st July)**. S = suppressed (<6). IDI confidentiality rules require for all counts to be randomly rounded up or down to the next multiple of 3 and percentages calculated from the rounded counts. Therefore, the numbers in each sub-group may not add up to the exact overall number that these sub-groups were derived from.

**Figure 2:**
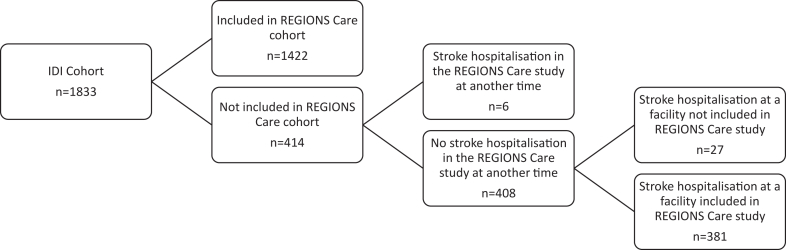
**Explanation of why people who are part of the IDI cohort did not appear in the REGIONS Care cohort as having a stroke during the same period (1st May and 31st July)**. S = suppressed (<6). IDI confidentiality rules require for all counts to be randomly rounded up or down to the next multiple of 3 and percentages calculated from the rounded counts. Therefore, the numbers in each sub-group may not add up to the exact overall number that these sub-groups were derived from.

Among the 300 cases missed by the IDI, 102 had a stroke recorded only as a secondary administrative diagnosis, 24 were recorded as transferred from another hospital facility instead of a first admission and 174 had no record of a stroke hospitalisation between May and July 2018 (Fig. 1). Among the 414 ‘false-positive’ cases, 6 patients were collected in the REGIONS Care study but outside of the May–July period (Fig. 2). Among the remaining 408 cases who were not collected in REGIONS Care, 27 were recorded in the IDI as admitted in a facility not included in the REGIONS care study.

The detailed hospital medical record audit at two centres by stroke experts showed that of the 43 original ‘false-positives’ in the audited urban thrombectomy centre, 33/43 (77%) did have a primary stroke diagnosis, and of 24 ‘false-positives’ in the audited non-urban primary stroke centre, 10/24 (42%) had a confirmed primary stroke diagnosis (Supplementary TableS5). The most common reason for missing these patients in REGIONS Care were: patients not being referred to the stroke services, dying shortly after admission, or gaps in stroke clinical nurse specialist cover during periods of leave meaning interrupted case ascertainment. Among cases ‘mis-coded’ as strokes in the administrative data, true ‘false positives’ most represented stroke mimics (e.g. tumours, migraine, falls, confusion), TIAs, or old strokes admitted with a stroke complication or other primary acute medical issue. When sensitivity and PPV were recalculated for these two hospitals using the updated counts, IDI data from the urban thrombectomy centre had a sensitivity of 92% (up from 88%) and a PPV of 91% (up from 62%), and the non-urban primary stroke centre a sensitivity of 80% (up from 77%) and PPV of 80% (up from 66%), suggesting that the PPV for the overall study was originally underestimated.

### Stroke risk factors

Overall, 1926 patients were included in both REGIONS Care and IDI Cohort 2 during the period 1 May-31 October 2018. Comparisons between IDI and REGIONS Care measurements for stroke risk factors are shown in Table 3. For diabetes, atrial fibrillation, prior stroke and regular smoking (see Supplementary Table S1 for definitions), sensitivities ranged from 71% to 93%, specificities from 90% to 97%, and kappa-statistics from 0.69 to 0.78. The proportion of false-positives (patients wrongly coded as presenting a risk factor in the IDI) was high for hypertension and dyslipidaemia, with specificities of 61% and 52%, respectively but the proportion of false-negatives was low for these two risk factors (sensitivities of 91% and 85%). Identification of prior TIAs in the IDI had a sensitivity of 53% (Table 3).

**Table 3:** Comparison of stroke risk factors measurement in the IDI and in the REGIONS Care study among stroke patients identified in both the IDI and the REGIONS study for the period 1 May 2018–31 October 2018.

Risk factor	True-positive	False-negative	False-positive	True-negative	Kappa	Sensitivity (95% CI)	Specificity (95% CI)
Hypertension	1176	120	237	363	0.54 (0.5,0.58)	91 (89, 92)	61 (57,64)
Dyslipidaemia	591	105	543	600	0.33 (0.3,0.37)	85 (82, 88)	52 (50, 55)
Diabetes	414	33	132	1308	0.78 (0.7,0.8)	93 (90, 95)	91 (89, 92)
Atrial fibrillation	474	69	129	1200	0.75 (0.72,0.78)	87 (84, 90)	90 (89, 92)
Prior stroke	249	99	66	1455	0.69 (0.65,0.74)	72 (67, 76)	96 (95,97)
Prior transient ischemic attack	123	111	66	1521	0.53 (0.47,0.59)	53 (46, 59)	96 (95, 97)
Regular smoker	156	63	48	1500	0.71 (0.66,0.76)	71 (65, 77)	97 (96, 98)

IDI confidentiality rules require for all counts to be randomly rounded up or down to the next multiple of 3 and percentages calculated from the rounded counts. Therefore, total numbers vary slightly between tables and may not add to 100%. Statistical tests were performed on the unrounded counts.

### Post-stroke outcomes

Comparisons for post-stroke outcomes are presented in Table 4. Overall, 1032 cases had an mRS 0–3 at 3 months post-stroke and 903 of them were classified as having a favourable outcome in the IDI (sensitivity 88%, specificity 82%), with substantial agreement between both measures (kappa = 0.67). When restricting analyses to patients alive at 3 months, the specificity decreased to 49%, suggesting that “alive” vs. “dead” was the strongest driver of the composite variable “favourable outcome”; sensitivity did not change. Agreement between the measures of being alive in REGIONS Care and the IDI was substantial (kappa = 0.91). Agreement was moderate for the “working” component (kappa = 0.33), with a low specificity of 32% and moderate for the “at the same address” component (kappa = 0.51). The sensitivity of the IDI to capture recurrent strokes within 3 months was low (27%), while the specificity was high (97%). The sensitivity and specificity for capturing readmissions to hospital were both high (82% and 86%, respectively). Hospital length of stay was associated with SSV and mRS at discharge (both p < 0.0001) (Supplementary Table S6), but explained variance was <10% for each. Including rehabilitation in ‘length of stay’ did not alter results (data not shown).

**Table 4:** Comparison of post-stroke outcomes at 3 months between IDI and REGIONS Care for strokes occurring in period 1 May 2018–31 October 2018.

Outcome (three months)	True-positive^a^	False-negative^a^	False-positive^a^	True-negative^a^	Kappa	Sensitivity (95% CI)	Specificity (95% CI)
mRS (0–3 vs. 4–6) vs. IDI favourable outcome	903	129	84	375	0.67 (0.63,0.71)	88 (85,90)	82 (78, 85)
mRS (0–3 vs. 4–5) vs. IDI favourable outcome^a^	903	129	54	51	0.27 (0.19,0.34)	87 (85, 89)	49 (39, 58)
Alive at 3 months	1137	S	48	309	0.91 (0.88,0.93)	S	87 (83,90)
Working at 3 months^b^	1347	30	51	24	0.33 (0.22,0.44)	98 (97, 99)	32 (21, 43)
At the same address at 3 months^b^	984	78	33	75	0.51 (0.43,0.59)	93 (91, 94)	69 (61, 78)
Recurrent stroke at 3 months^b^	9	24	33	1056	0.25 (0.12,0.38)	27 (12, 42)	97 (96, 98)
Readmission to hospital at 3 months^b^^,^^c^	153	33	129	813	0.57 (0.51,0.62)	82 (77, 88)	86 (84, 89)

S = suppressed (<6).

IDI confidentiality rules require for all counts to be randomly rounded up or down to the next multiple of 3 and percentages calculated from the rounded counts. Therefore, total numbers vary slightly between tables and may not add to 100%. Statistical tests were performed on the unrounded counts.

a Patients alive at 3 months only, i.e. mRS = 0–5.

b Patients alive at 3 months, i.e. mRS = 0–5 or with missing mRS.

c Excluding all readmissions for rehabilitation in IDI.

### Post-stroke interventions

Medication prescription information obtained at 3 months post-stroke in the IDI correctly identified 82% of the anticoagulant prescriptions, 81% of the antihypertensive medication prescriptions, 94% of the statin prescriptions and 91% of the antiplatelet prescriptions collected at discharge in REGIONS Care (Table 5). Specificities were also high for all medications, showing the reliability of the IDI to correctly identify post-stroke prescriptions. Measurement of thrombectomy in the IDI had a sensitivity of 88% and a kappa value of 0.91.

**Table 5:** Comparison of post-stroke interventions between IDI (three months post-stroke) and REGIONS Care (hospital discharge) for strokes occurring in period 1 May 2018–31 October 2018.

Post-stroke intervention	True-positive	False-negative	False-positive	True-negative	Kappa	Sensitivity (95% CI)	Specificity (95% CI)
Anti-platelets prescription	1029	99	126	666	0.76 (0.73,0.79)	91 (90,93)	84 (82,87)
Statins prescription	1110	72	126	606	0.78 (0.75,0.80)	94 (93,95)	83 (80,86)
Antihypertensive medications prescription	960	228	165	570	0.58 (0.54,0.61)	81 (79,83)	78 (75,81)
Anticoagulants prescription	342	75	132	1368	0.7 (0.66,0.74)	82 (78,86)	91 (90,93)
Thrombectomy	45	6	S	1866	0.91 (0.85,0.97)	88 (79,97)	S

S = suppressed (<6).

IDI confidentiality rules require for all counts to be randomly rounded up or down to the next multiple of 3 and percentages calculated from the rounded counts. Therefore, total numbers vary slightly between tables and may not add to 100%. Statistical tests were performed on the unrounded counts.

## Discussion

This study has shown that administrative data in a well-developed healthcare system with established data systems provides a valid and efficient alternative to manual, clinician-collected stroke data. The IDI dataset had a sensitivity of 83% and a PPV of 77% for stroke case ascertainment. There was no evidence of systematic bias in misidentification of stroke cases by ethnicity, sex and age. Also, 95% confidence limits around sensitivity and PPV values were relatively narrow suggesting a relatively high level of certainty in these estimates. Although the PPV is dependent on the prevalence of stroke and may therefore vary between populations (dependent on the true prevalence in each of those populations) it is not unreasonable to make comparisons with other studies that used similar approaches, including a systematic review of studies that validated ICD codes for stroke ascertainment in administrative datasets. This study found that the sensitivity of sets of stroke-specific codes was ≥82% in 13 of 22 studies where this was reported (range 34%–97%) and the PPV was ≥86% in 18 of the 34 studies that reported this statistic (range 32%–98%).^3^ Our results are therefore very similar, particularly when taking into account adjustments for missed cases in REGIONS Care as identified by the hospital medical record audit.

Positive predictive value for ‘unspecified’ stroke (I64) was low. Most (>70%) of the false positive I64 patients identified as stroke in REGIONS Care were reported as cerebral infarction (I63) in REGIONS Care (exact percentage not available due to Statistics New Zealand confidentiality requirements). A likely reason for this is that clinicians frequently do not specify ‘infarction’ or ‘ischaemia’ when they document ‘stroke’ and hospital coding teams are not able to use subtle ancillary data (such as a normal early CT supporting cerebral infarction and excluding ICH) when making coding allocations; in contrast, clinicians entering registry data are able to consider such imaging findings for allocation into the clinical registry. This issue highlights the importance of educational programs to improve clinical documentation to facilitate accurate coding.^11^

Similar to other studies,^4^^,^^12^ we found that administrative data identification of diabetes and atrial fibrillation for hospitalised stroke patients was good. However, our IDI-algorithm identified more people as having hypertension and dyslipidaemia were identified to have these conditions during clinician review, resulting in low specificity (i.e. the algorithm did not correctly classify people who did not have the risk factor); however, sensitivity was high (i.e. the algorithm performed well at classifying people who did have the risk factor). Prior studies have found underreporting of hypertension and dyslipidaemia in administrative datasets compared with medical chart reviews,^4^^,^^13^ which contrasts with our results. However, these studies only used hospitalisation database ICD diagnosis codes; our algorithm also included pharmaceutical data, which likely led to overreporting. Our diabetes and atrial fibrillation IDI algorithms also included pharmaceuticals, however, this did not lead to overreporting, presumably because medications prescribed for these risk factors are not as frequently used for other conditions. Prior strokes were captured well by the IDI, but prior TIAs were poorly identified. We did not include incident TIA cases in REGIONS Care so did not validate the capture of TIA other than as a risk factor, however, other studies have also found administrative data captures TIA more poorly than acute stroke,^5^^,^^14^ likely because many TIAs are no longer hospitalised and diagnosing TIA can be challenging. Lastly, smoking status had very high specificity (97%) and lower sensitivity (71%). Smoking status was obtained from (self-reported) census data, which may explain the good agreement. Census data can be problematic if collected a long time before or after the study. However, for our study, the date of census (6th March 2018) was very close to the study period (1st May to 31st July 2018). Multiple algorithms using different data sources and methods have been proposed to ascertain smoking status in electronic health data, but many had low sensitivity and positive predictive value.^15^

The post-stroke favourable outcome algorithm at 3 months post-stroke had good agreement with mRS 0–3 at 3 months. However, when we restricted the analyses to only patients still alive at 3 months, the IDI algorithm classified some people as having a favourable outcome who in fact had a mRS score of >3 at 3 months. The poorer performance of the employment and address change components were also observed when validating them individually. In contrast, other studies have found that ‘home time’ (total number of days a patient is living outside of a healthcare institution after stroke) correlates with mRS,^16^, ^17^, ^18^ as well as other measures of functional status after stroke.^19^ The performance of our score may be enhanced if using a continuous instead of dichotomous measure as was done in other studies.

We found that length of stay was associated with stroke severity in REGIONS Care, meaning it could potentially be used as a proxy for severity, at least in epidemiological studies. Different measures of stroke severity using administrative data developed by others have also shown good agreement with clinically defined severity.^19^^,^^20^

We found that the IDI was able to accurately identify thrombectomy and post-stroke prescriptions. Few studies have previously assessed the accuracy of thrombectomy recording in administrative datasets, but one US study found that MS-DRG billing codes were necessary to be included because ICD codes alone missed 13% of thrombectomies.^21^ Previous research has reported mixed results on how well thrombolysis is captured by administrative data,^21^, ^22^, ^23^ but this could not be validated in our study because it only started being coded in New Zealand after the study period. The finding that post-stroke prescription use is highly accurate using administrative data is of special utility as many registries currently do not routinely capture these data, yet it is critical to assess secondary prevention.

National stroke registries can lead to improvements in the quality of care, patient outcomes, and health policy^24^ and enable assessment of whether patient outcomes are equitable. Traditional registries require resource intensive and ongoing data collection by clinicians at all acute stroke hospitals as well as central oversight. Administrative data provides a lower cost alternative that has several advantages: less pressure on clinical staff to continually report data into a register; data may be more reliable using structured and automated extraction from central administrative data compared with manual extraction by a variety of people across hospitals; and there may be less bias. There are also disadvantages, including: timeliness of data availability; potential linkage errors, which may result in misclassification and bias, and incomplete community case-ascertainment meaning non-hospitalised stroke cases may be missed, thus potentially affecting incidence estimates. The latter is, of course, also a limitation of hospital-based registries and other data sources are required to validate NZ administrative data against population-based incidence data, which was not the purpose of this study. An additional limitation of administrative data is that some key information is unavailable in administrative data, including: mRS; door to needle time; stroke unit care; swallow screen; early mobilisation; and data on patient satisfaction, or other more patient-focussed measures. Therefore, despite its apparent validity regarding hospital stroke case ascertainment and many other key variables, a virtual register would require at least some data supplementation to achieve a comprehensive care overview; a hybrid or combined model likely represents the ideal solution. A supplementary stroke care register to purposefully collect additional patient survey data would thus likely add significant value. Increasingly, electronic clinical records allow automated extraction from notes to databases providing further efficiency gains, which may offer low resource intense solutions to address some of these gaps.

This study had some limitations. Firstly, REGIONS Care included all hospitalised strokes for the first 3 months, after which some of the larger hospitals had reached their minimum sample size, so ceased capturing new strokes. Despite this, the first 3 months sample size was large enough to complete validation of case ascertainment, and for the validation of risk factors, outcomes and interventions we were able to include the whole dataset (6 months). Secondly, REGIONS Care had some missing cases, meaning the dataset on which we made comparisons for case ascertainment was not entirely complete. An audit of hospital medical records in two centres suggests that our estimates of sensitivity and particularly PPV for the whole study population were thus slightly underestimated, so IDI-based estimates of stroke incidence are likely more reliable than presented here. Thirdly, this study is not generalisable to all settings. In particular, our findings are based on a well-developed healthcare system in a high-income country with a small population with well-established data systems. The validity of administrative datasets in other countries to identify hospitalised stroke cases, risk factors, outcomes, and interventions therefore may not be the same.

In conclusion, we found that administrative data within the context of a well-developed healthcare system with established data systems provides a valid and efficient alternative to manual, clinician-collected hospital-based stroke data. Therefore, while some manual data collection will continue to provide additional value in areas not currently well-captured by administrative data, overall the development of an administrative national virtual stroke register to track hospitalised stroke patients and service performance for health service planning in NZ appears feasible and useful.

## Contributors

Conceptualisation: AR and JD; Funding acquisition: AR, JD, SGT, MH, AD, JNF, PAB, JHG, DAC, HM, VLF, AR; Data management and analysis: MC and HJD; Manuscript Writing: MC, HJD, JD, MW, SGT, MH, AD, JNF, PAB, JHG, DAC, WML, HM, JK, VLF, AR. MC and HJD contributed equally as co-first authors.

## Data sharing statement

Following Statistics NZ confidentiality requirements, data are only available to approved researchers in secure Statistics NZ datalabs.

## Declaration of interests

The following authors declare potential conflicts of interest: P. Alan Barber—Neurological Foundation of New Zealand supports a personal chair in clinical neurology, DSMB: 2023–2024 EMView Study, President of the Australia and New Zealand Association of Neurologists; William Levack—Royalties from Francis & Taylor paid to him for a book on “Rehabilitation Goal Setting,” published in 2014; Honoraria for guest lecture on a topic unrelated to this manuscript at St Luke’s International University, Tokyo, Osaka Metropolitan University, Osaka, and Showa University, Tokyo, and salary from University of Otago; Marine Corbin—co-investigator on several unrelated grants including from Health Research Council New Zealand, Lotteries New Zealand, and Ministry of Business, Innovation, and Employment: A better Start National Challenge. The other authors declared no potential conflicts of interest with respect to the research, authorship, and/or publication of this article beyond Health Research Council funding for this project indicated under ‘Funding’ above.
