# Supplementary material for: Can administrative data be used for a national register of hospitalised stroke patients? A New Zealand validation study
Source: Lancet Reg Health West Pac. 2026 Jan 7;66:101768. doi: 10.1016/j.lanwpc.2025.101768 (PMC12809099; doi:10.1016/j.lanwpc.2025.101768)
Supplement: Supplemental — Tables [file mmc1.docx]

**Supplementary Tables**

| **Table S1. Definitions of IDI created variables** | | | |
| --- | --- | --- | --- |
|  |  |  |  |
| **Variable** | **Datasets** | **Codes or Criteria** | **Details** |
| Stroke | National Minimum Dataset (NMDS): Publicly funded hospital discharges – diagnosis/procedure information | ICD-10-AM I61 Intracerebral haemorrhage | First stroke within study dates (IDI Cohort 1: 1 May-31 July 2018; IDI Cohort 2: 1 May-31 October 2018) |
|  |  | ICD-10-AM I63 Cerebral infarction |  |
|  |  | ICD-10-AM I64 Stroke unspecified |  |
| Hypertension | National Minimum Dataset (NMDS): Publicly funded hospital discharges – diagnosis/procedure information | ICD-10-AM I10, ICD-9-CMA 401 Essential (primary) hypertension | At anytime prior to study stroke date |
|  |  | ICD-10-AM I12, ICD-9-CMA 403 Hypertensive chronic kidney disease |  |
|  | Pharmaceutical data | 1061 Losartan Potassium | Two or more dispensings within the 10 years prior to study stroke. |
|  |  | 1116 Bendrofluazide |  |
|  |  | 1127 Cilazapril with hydrochlorothiazide |  |
|  |  | 1254 Candesartan cilexetil |  |
|  |  | 2398 Felodipine |  |
|  |  | 2708 Enalapril maleate with hydrochlorothiazide |  |
|  |  | 2711 Enalapril maleate |  |
|  |  | 2770 Cilazapril |  |
|  |  | 2772 Quinapril |  |
|  |  | 2793 Amlodipine |  |
|  |  | 2794 Benazepril |  |
|  |  | 2795 Lisinopril with hydrochlorothiazide |  |
|  |  | 2797 Lisinopril |  |
|  |  | 2806 Perindopril |  |
|  |  | 2840 Captopril with hydrochlorothiazide |  |
|  |  | 2841 Captopril |  |
|  |  | 3749 Quinapril with hydrochlorothiazide |  |
| Dyslipidemia | National Minimum Dataset (NMDS): Publicly funded hospital discharges – diagnosis/procedure information | ICD-10-AM E780, ICD-9-CMA 2720 Pure hypercholesterolemia | At anytime prior to study stroke date |
|  |  | ICD-10-AM E785, ICD-9-CMA 2724 Hyperlipidemia, unspecified |  |
|  | Pharmaceutical data | 1063 Fluvastatin | Two or more dispensings within the 10 years prior to study stroke. |
|  |  | 1137 Atorvastatin |  |
|  |  | 2513 Simvastatin |  |
|  |  | 2780 Pravastatin |  |
|  |  | 3853 Ezetimibe |  |
|  |  | 3799 Ezetimibe with simvastatin |  |
| Diabetes^1^ | National Minimum Dataset (NMDS): Publicly funded hospital discharges – diagnosis/procedure information | ICD-10-AM E10 Type 1 diabetes mellitus | At anytime prior to study stroke date |
|  |  | ICD-10-AM E11 Type 2 diabetes mellitus |  |
|  |  | ICD-10-AM E12 Malnutrition-related diabetes mellitus |  |
|  |  | ICD-10-AM E13 Other specified diabetes mellitus |  |
|  |  | ICD-10-AM E14 Unspecified diabetes mellitus |  |
|  |  | ICD-10-AM O240 Pre-existing diabetes mellitus, Type 1, in pregnancy |  |
|  |  | ICD-10-AM O241 Pre-existing diabetes mellitus, Type 2, in pregnancy |  |
|  |  | ICD-10-AM O242 Pre-existing diabetes mellitus, other specified type, in pregnancy |  |
|  |  | ICD-10-AM O243 Pre-existing diabetes mellitus, unspecified, in pregnancy |  |
|  |  | ICD -9-CMA 250 Diabetes mellitus |  |
|  |  | Note: Admissions with a code for gestational diabetes are not included. | |
|  | Pharmaceutical data | 1192 Insulin lispro | Two or more dispensings in any two year period |
|  |  | 1247 Acarbose |  |
|  |  | 1567 Glibenclamide |  |
|  |  | 1568 Gliclazide |  |
|  |  | 1569 Glipizide |  |
|  |  | 1648 Insulin neutral |  |
|  |  | 1649 Insulin isophane |  |
|  |  | 1655 Insulin zinc suspension |  |
|  |  | 1794 Metformin hydrochloride |  |
|  |  | 2276 Tolazamide |  |
|  |  | 2277 Tolbutamide |  |
|  |  | 3739 Rosiglitazone |  |
|  |  | 3783 Insulin aspart |  |
|  |  | 3800 Pioglitazone |  |
|  |  | 3857 Insulin glargine |  |
|  |  | 3882 Insulin lispro with insulin lispro protamine |  |
|  |  | 3908 Insulin glulisine |  |
|  |  | 3982 Insulin aspart with insulin aspart protamine |  |
|  |  | 6300 Insulin isophane with insulin neutral |  |
|  |  | Note: metformin is also used to treat polycystic ovary syndrome in women aged 12-45. Women within this age group, dispensed metformin and with no other record of diabetes through diagnose, laboratory tests, outpatient visits or other medication, have not been included. Likewise, insulin is also used to treat gestational diabetes and therefore women dispensed insulin around the time of birth have not been included. | |
|  | National Non-Admitted Patient Collection (NNAPAC) | M20006 Diabetes “education and management” | At anytime prior to study stroke date |
|  |  | M20007 Diabetes retinal (fundus) screening |  |
|  | Laboratory claims data | BG2 Glycosylated haemoglobin (HbA1c) | Four or more tests in any two year period prior to study stroke date |
|  |  | BP8 Urine albumin to creatinine ratio (ACR) test | Two or more tests in any two year period prior to study stroke date |
| Atrial fibrillation | National Minimum Dataset (NMDS): Publicly funded hospital discharges – diagnosis/procedure information | ICD-10-AM I48, ICD-9-CMA 4273 Atrial fibrillation and flutter | At anytime prior to study stroke date |
|  | Pharmaceutical data | 2331 Warfarin sodium | Two or more dispensings within the 10 years prior to study stroke. |
|  |  | 3937 Dabigatran |  |
|  |  | 3924 Rivaroxiban |  |
| Regular smoker | Census 2018 | Obtained from census regular smoker dichotomous variable from the question “Do you smoke cigarettes regularly (that is, one or more a day)? | At Census 2018 (6th March 2018). |
| Prior stroke | National Minimum Dataset (NMDS): Publicly funded hospital discharges – diagnosis/procedure information | ICD-10-AM I61 Intracerebral haemorrhage | At anytime prior to study stroke date |
|  |  | ICD-10-AM I63 Cerebral infarction |  |
|  |  | ICD-10-AM I64 Stroke unspecified |  |
| Prior TIA | National Minimum Dataset (NMDS): Publicly funded hospital discharges – diagnosis/procedure information | ICD-10-AM G45 Transient cerebral ischaemic attacks and related syndromes | At anytime prior to study stroke date |
| Thrombectomy | National Minimum Dataset (NMDS): Publicly funded hospital discharges – diagnosis/procedure information | Procedure codes:  35414-00, 90235-00 Embolectomy or thrombectomy of intracranial artery  90230-00 Embolectomy or thrombectomy of other artery | Within three months of study stroke date |
| Post-stroke anticoagulant medication | Pharmaceutical data | 2331 Warfarin sodium | Dispensed within three months of stroke |
|  |  | 3937 Dabigatran |  |
|  |  | 3924 Rivaroxiban |  |
| Post-stroke hypertension medication | Pharmaceutical data | 1061 Losartan Potassium | Dispensed within three months of stroke |
|  |  | 1116 Bendrofluazide |  |
|  |  | 1127 Cilazapril with hydrochlorothiazide |  |
|  |  | 1254 Candesartan cilexetil |  |
|  |  | 2398 Felodipine |  |
|  |  | 2708 Enalapril maleate with hydrochlorothiazide |  |
|  |  | 2711 Enalapril maleate |  |
|  |  | 2770 Cilazapril |  |
|  |  | 2772 Quinapril |  |
|  |  | 2793 Amlodipine |  |
|  |  | 2794 Benazepril |  |
|  |  | 2795 Lisinopril with hydrochlorothiazide |  |
|  |  | 2797 Lisinopril |  |
|  |  | 2806 Perindopril |  |
|  |  | 2840 Captopril with hydrochlorothiazide |  |
|  |  | 2841 Captopril |  |
|  |  | 3749 Quinapril with hydrochlorothiazide |  |
| Post-stroke statin medication | Pharmaceutical data | 1063 Fluvastatin | Dispensed within three months of stroke |
|  |  | 1137 Atorvastatin |  |
|  |  | 2513 Simvastatin |  |
|  |  | 2780 Pravastatin |  |
|  |  | 3853 Ezetimibe |  |
|  |  | 3799 Ezetimibe with simvastatin |  |
| Post-stroke antiplatelet medication | Pharmaceutical data | 1087 Aspirin | Dispensed within three months of stroke |
|  |  | 1246 Clopidogrel |  |
|  |  | 3860 Clopidogrel |  |
| Recurrent stroke | National Minimum Dataset (NMDS): Publicly funded hospital discharges – diagnosis/procedure information | ICD-10-AM I61 Intracerebral haemorrhage | Within three months of study stroke date |
|  |  | ICD-10-AM I63 Cerebral infarction |  |
|  |  | ICD-10-AM I64 Stroke unspecified |  |
| Readmission to hospital | National Minimum Dataset (NMDS): Publicly funded hospital discharges – diagnosis/procedure information |  | Any readmission to hospital within three months of study stroke date, not including same-day transfers |
| Length of stay | National Minimum Dataset (NMDS): Publicly funded hospital discharges – diagnosis/procedure information |  | Includes transfers where the start date of a subsequent hospital stay is the same as the end date of a previous stay |
| IDI stroke favourable outcome | Department of Internal Affairs (DIA) Deaths data |  | Favourable outcome if no death, job loss or address change within three months post study stroke date |
|  | Inland Revenue Department (IRD) Employee Monthly Schedule (EMS) table subset |  |  |
|  | Address notification data |  |  |

| **Table S2. Formulas for asymptotic 95% confidence intervals of Sensitivity, Specificity and Positive predictive value** | | | |
| --- | --- | --- | --- |
|  | Standard error (SE) | 95% lower confidence limit | 95% upper confidence limit |
| Sensitivity | √[Sensitivity*(1-Sensitivity)/(TP+FN)] | Sensitivity -1.96*SE (Sensitivity) | Sensitivity +1.96*SE (Sensitivity) |
| Positive Predictive Value (PPV) | √[PPV*(1-PPV)/(TP+FP)] | PPV -1.96*SE (PPV) | PPV +1.96*SE (PPV) |
| Specificity | √[Specificity*(1-Specificity)/(TN+FP)] | Specificity -1.96*SE (Specificity) | Specificity +1.96*SE (Specificity) |

| **Table S3. Number of stroke cases identified in the IDI and in the REGIONS Care study for the period 1 May 2018-31 July 2018 by stroke type, sex and age group** | | | | | | | |
| --- | --- | --- | --- | --- | --- | --- | --- |
|  | **Identified in IDI** | **Identified in REGIONS** | **True-positive** | **False-negative** | **False-positive** | **Sensitivity (95%CI)** | **Positive Predictive Value (95%CI)** |
| **Stroke type** |  |  |  |  |  |  |  |
| **ICH** | 237 | 219 | 153 | 66 | 84 | 70 (64,76) | 65 (58,71) |
| **Cerebral Infarction** | 1494 | 1395 | 1122 | 273 | 372 | 80 (78,83) | 75 (73,77) |
| **Unspecified Stroke** | 102 | 102 | 18 | 87 | 84 | 17 (10,24) | 18 (10,25) |
| **Sex** |  |  |  |  |  |  |  |
| **Males** | 906 | 882 | 723 | 159 | 183 | 82 (79,85) | 80 (77,82) |
| **Females** | 927 | 837 | 696 | 138 | 228 | 83 (81,86) | 75 (73,78) |
| **Age group** |  |  |  |  |  |  |  |
| **Under 60** | 276 | 267 | 222 | 42 | 51 | 84 (80,89) | 81 (77,86) |
| **60 and over** | 1560 | 1452 | 1200 | 252 | 360 | 83 (81,85) | 77 (75,79) |
| IDI confidentiality rules require for all counts to be randomly rounded up or down to the next multiple of 3 and percentages calculated from the rounded counts. Therefore, total numbers vary slightly between tables and may not add to 100%. Statistical tests were performed on the unrounded counts. | | | | | | | |

| **Table S4. DHB of domicile of people with a stroke hospitalisation in the IDI between May to July 2018 and all people in the REGIONS Care study May to July 2018** | | |
| --- | --- | --- |
| DHB of Domicile | **IDI cohort**  **(N = 1833)** | **REGIONS cohort**  **(N = 1719)** |
| Northland | 102 (5.6%) | 93 (5.4%) |
| Waitemata | 162 (8.8%) | 165 (9.6%) |
| Auckland | 123 (6.7%) | 87 (5.1%) |
| Counties Manukau | 180 (9.8%) | 183 (10.6%) |
| Waikato | 171 (9.3%) | 156 (9.1%) |
| Lakes | 54 (2.9%) | 57 (3.3%) |
| Bay of Plenty | 90 (4.9%) | 84 (4.9%) |
| Tairawhiti | 21 (1.1%) | 15 (0.9%) |
| Hawke's Bay | 78 (4.3%) | 63 (3.7%) |
| Taranaki | 51 (2.8%) | 45 (2.6%) |
| Midcentral | 78 (4.3%) | 69 (4.0%) |
| Whanganui | 24 (1.3%) | 36 (2.1%) |
| Capital and Coast | 114 (6.2%) | 114 (6.6%) |
| Hutt | 60 (3.3%) | 54 (3.1%) |
| Wairarapa | 21 (1.1%) | 15 (0.9%) |
| Nelson Marlborough | 63 (3.4%) | 51 (3.0%) |
| West Coast | 12 (0.7%) | 12 (0.7%) |
| Canterbury | 255 (13.9%) | 261 (15.2%) |
| South Canterbury | 21 (1.1%) | 21 (1.2%) |
| Southern | 153 (8.3%) | 132 (7.7%) |
| IDI confidentiality rules require for all counts to be randomly rounded up or down to the next multiple of 3 and percentages calculated from the rounded counts. Therefore, total numbers vary slightly between tables and may not add to 100%. Statistical tests were performed on the unrounded counts. | | |

**Table S5. Discharge summary audit results**

| Hospital | Identified in administrative data (NMDS) | Identified in REGIONS | Original True-Positive | Original False-Positive | False-Negatives | Wrong False-Positive (Missed by REGIONS Care) | Original sensitivity | Original PPV | Final True-Positive | Final False-positive | Final Sensitivity | Final PPV |
| --- | --- | --- | --- | --- | --- | --- | --- | --- | --- | --- | --- | --- |
| Non-urban primary stroke centre | 71 | 61 | 47 | 24 | 14 | 10 | 77 (66,88) | 66 (55,77) | 57 | 14 | 80 (71,89) | 80(71,89) |
| Urban thrombectomy centre | 112 | 78 | 69 | 43 | 10 | 33 | 88 (81,95) | 62 (53,71) | 102 | 10 | 92 (87,97) | 91 (86,96) |
| IDI confidentiality rules require for all counts to be randomly rounded up or down to the next multiple of 3 and percentages calculated from the rounded counts. Therefore, total numbers vary slightly between tables and may not add to 100%. Statistical tests were performed on the unrounded counts. | | | | | | | | | | | | |

NMDS = National Minimum Dataset
This sub-analysis is limited to patients domiciled in the respective DHB and who presented to the respective DHB to ensure datasets match - therefore counts may not match other analyses

| **Table S6. Association between stroke severity (SSV, mRS at discharge) collected in REGIONS and hospital length of stay measured in IDI for strokes occurring in period 1 May 2018-31 October 2018** | | | |
| --- | --- | --- | --- |
|  |  | **Length of stay** |  |
|  | **N** | **median (1st quartile, 3rd quartile)** | **p-value for Kruskal-Wallis test** |
| **Six simple variable (SSV)^α^ (missing=S)** |  |  |  |
| **3** ^a^* | 636 | 3 (2,5) | <0.0001 |
| **2** ^b^ | 477 | 5 (3,7) |  |
| **1** ^b^ | 396 | 6 (3,9) |  |
| **0** ^b^ | 411 | 5 (3,9) |  |
|  |  |  |  |
| **Modified Rankin Scale (mRS) at discharge (missing=12)** |  |  |  |
| **0** ^a^ | 261 | 3 (2,5) | <0.0001 |
| **1** ^a^ | 420 | 3 (2,5) |  |
| **2** ^b^ | 357 | 4 (3,7) |  |
| **3** ^c^ | 336 | 6 (4,9) |  |
| **4** ^c^ | 210 | 7 (4,11) |  |
| **5** ^c^ | 126 | 7 (4,14) |  |
| **6** ^b^ | 201 | 5 (2,8) |  |
| S = suppressed (<6)  IDI confidentiality rules require for all counts to be randomly rounded up or down to the next multiple of 3 and percentages calculated from the rounded counts. Therefore, total numbers vary slightly between tables and may not add to 100%. Statistical tests were performed on the unrounded counts. | | | |

^α^Comprises the variables: age; living alone pre-stroke; pre-stroke functional status; normal verbal Glasgow Coma Scale score; ability to lift arms; ability to walk unaided.

*Dwass, Steel,Critchlow-Flligner (DSCF) multiple comparison analysis. For each variable (SSV or mRS), values that do not share the same superscript are associated with statistically significantly (p<0.05) different lengths of stay.
